# Cefiderocol-Resistant *Elizabethkingia anophelis* Bacteremia Following WATCHMAN Implantation: A Case Report and Review of the Literature

**DOI:** 10.1155/crdi/5221364

**Published:** 2025-05-20

**Authors:** Anna DeFrank, Michael Bosco, Sigridh Muñoz Gómez, Amit Dhaliwal

**Affiliations:** ^1^Department of Pharmacy, NYU Langone Hospital – Long Island, Mineola, New York, USA; ^2^Department of Medicine, Division of Infectious Disease, NYU Grossman Long Island School of Medicine, Mineola, New York, USA

**Keywords:** case report, cefiderocol, *Elizabethkingia*, endocarditis, resistance

## Abstract

*Elizabethkingia anophelis* is an emerging pathogen associated with nosocomial and community outbreaks. Treatment of infection caused by *E. anophelis* is not well-defined given its extensive drug resistance profile, and infection carries a poor prognosis. *E. anophelis* is intrinsically resistant to many classes of antibiotics including carbapenems and polymyxins due to multiple resistance genes. Resistance to novel β-lactam/β-lactamase inhibitors has also been reported. The activity of cefiderocol (FDC) is unknown. Here, we describe a case of FDC-resistant *E. anophelis* bacteremia following WATCHMAN implantation. To our knowledge, this is the first reported case of FDC resistance amongst *Elizabethkingia* spp.

## 1. Introduction

*Elizabethkingia anophelis*, an environmental, aerobic, glucose-nonfermenting, oxidase-positive, Gram-negative bacilli [1], was first identified in 2011 from the *Anopheles gambiae* mosquito [2]. Since the first episode of human disease was reported [3], numerous nosocomial and community outbreaks have been reported worldwide [4–10]. Many of these cases were originally misidentified as *Elizabethkingia meningoseptica* using commercial identification systems (i.e., VITEK 2, MALDI-TOF) [11].

*E. anophelis* infections occur predominantly in patients at extremes of life or with underlying comorbidities such as chronic obstructive pulmonary disease, diabetes, unhealed wounds, and ventilator-dependence. Common disease manifestations include pneumonia (PNA), catheter-related bacteremia, and neonatal meningitis, with mortality ranging from 18% to 70% [11–13].

*E. anophelis* is intrinsically resistant to many classes of antibiotics including carbapenems due to multiple chromosomally encoded β-lactamases: *bla*_B_ and *bla*_GOB_, two metallo-β-lactamases (MBLs); *bla*_CME_, a Class D serine β-lactamase [14, 15]. Resistance to non-*β*-lactam agents including fluoroquinolones, tetracyclines, aminoglycosides, and newer *β*-lactam/*β*-lactamase inhibitors has also been reported [1]. The activity of cefiderocol (FDC), a novel siderophore cephalosporin, against *Elizabethkingia* spp. is relatively unknown given its recent approval and limited number of cases reported. To date, FDC resistance amongst *Elizabethkingia* spp. has not been reported.

The WATCHMAN device is a small, parachute-shaped device that is implanted in the left atrial appendage of the heart to seal it off. It is used as an alternative to anticoagulation in selected patients to prevent strokes. While rare, infections are a potential complication associated with the WATCHMAN device.

## 2. Case Presentation

An 82-year-old patient presented with acute fever (103°F), nausea, vomiting, leukocytosis (13.6 × 10^9^ cells/L), and elevated CRP (126 mg/L), immediately after elective cardioversion for atrial fibrillation. Blood cultures were obtained and empiric vancomycin (VAN) and ceftriaxone (CRO) were initiated. Other comorbidities included chronic kidney disease, psoriasis, and recent right-knee prosthetic joint infection with *Pseudomonas aeruginosa* treated with surgical washout and a 6-month course of antibiotics (oral levofloxacin [LVX] plus minocycline). Six weeks prior to admission, he underwent atrial ablation with WATCHMAN implantation.

Both pairs of blood cultures on two consecutive days revealed *E. meningoseptica*. Antimicrobial susceptibility obtained by automated broth microdilution (BMD) showed resistance to all cephalosporins, including FDC, aminoglycosides, carbapenems, piperacillin/tazobactam (TZP), aztreonam (ATM), and tetracyclines. It was susceptible to fluoroquinolones and cotrimoxazole (SXT) (Table 1), and LVX 750 mg daily was initiated. The patient had no known prior exposure to FDC. The isolate was submitted to a reference lab for confirmatory testing and subsequent whole genome sequencing (WGS). Further workup revealed bibasilar opacities on CT chest. Sputum culture grew *Candida* species, and blood cultures repeated 48 h after antibiotic treatment remained negative. Given the propensity of *Elizabethkingia* spp. to form biofilms, transesophageal echocardiogram was performed to rule out device-related infection, which did not show any vegetation. He was treated with LVX for 2 weeks. At 6-week follow up, the patient was asymptomatic, chest X-ray showed resolution of lung opacities, two sets of blood cultures were negative, and his CRP had normalized.

## 3. Discussion

WGS identified *E. anophelis*, resistant to multiple antimicrobials, including FDC, with an MIC of 8 mcg/mL (Table 1) determined via BMD. The discrepancies in several MIC values may be secondary to known limitations with automated testing panels, whereas classic BMD allows for more precise values to be obtained [16]. For FDC specifically, reproducibility of MIC values may be a challenge given historic testing difficulties when utilizing iron-depleted cation-adjusted media [17]. To date, there are no reported cases of FDC resistance amongst *Elizabethkingia* spp. Mechanisms leading to reduced susceptibility or resistance to FDC amongst Gram-negative organisms have been evaluated in surveillance studies and case reports.

Treatment of *E. anophelis* infection is not well-defined given its extensive drug resistance profile and lack of standardized breakpoints for *Elizabethkingia* spp. Interpretations are inferred using CLSI breakpoints for *Enterobacterales* and lactose nonfermenters. *E. anophelis* exhibits a wide variation in susceptibility to TZP (0%–92%), ciprofloxacin (CIP) (1%–100%), LVX (16%–96%), and SXT (4%–70.6%) [18, 19]. Although clinical outcomes with monotherapy appear mixed, fluoroquinolone use and improved survival have been observed [20]. Improved survival utilizing dual therapy including either SXT, minocycline, rifampin, or a fluoroquinolone has been observed [6, 21, 22]. The role of newer β-lactam/β-lactamase inhibitors such as ceftazidime-avibactam (CZA), imipenem-relebactam (I-R) and meropenem-vaborbactam (MVB) against *E. anophelis* is not well defined.

A recent review by Koh et al. [23] found that the most common causes of *Elizabethkingia* spp. bacteremia were catheter-related (71%), followed by PNA (14%) and skin and soft tissue (1%). All of these were potential etiologies in our patient, although no etiology could be proved with certainty.

Several extended-spectrum β-lactamases have been identified in bacterial isolates with elevated FDC minimum inhibitory concentrations (MIC). Takemura et al. isolated PER, NDM, and VEB in *Acinetobacter baumannii* complex isolates, while NDM and VEB were isolated amongst Enterobacterales [24]. Tiseo et al. demonstrated that a KPC-3 variant with a D19Y substitution in a *Klebsiella pneumoniae* isolate led to FDC resistance [25]. Amongst *Enterobacter cloacae* complex isolates, deletions in the R2 loop of the AmpC β-lactamase were associated with FDC resistance [26]. In a study of five FDC-resistant *E. cloacae* isolates from a patient following prolonged FDC exposure, OXA-48 and NDM β-lactamases, and *cirA* gene mutation, which encodes for a siderophore iron transporter, were identified [27].

Disruptions in TonB-dependent siderophore uptake receptor genes can lead to loss of FDC transport into the bacterial cell. Iron transporter mutations in *pirA/D*, *cirA*, and *fiu* genes are documented in the literature [28]. Mutations in *ompR/envZ* and *exbD*, which affect iron transporter genes, may be associated with FDC resistance and were isolated in FDC-resistant *K. pneumoniae* strains by Yamano et al. [29]. These isolates also exhibited mutations in *baeS* and *yicM*, of which the clinical effects are unknown. Increased FDC MICs were observed amongst *P. aeruginosa* isolates following drug exposure with associated mutations in *fecA* (iron transporter-related gene) and *pvdS*, a regulator of pyoverdine synthesis [30].

OmpK35, OmpK36, and OmpK37 porin mutations in *K. pneumoniae* and OmpC and OmpF porin mutations in *Enterobacter* spp. have been detected in FDC-resistant strains [28]. Efflux pumps including sugE and chrA in *K. pneumoniae*, MexAB-OprM in *P. aeruginosa*, smeDEF in *Stenotrophomonas maltophilia*, and AxyABM in *Achromobacter xylosoxidans* are associated with elevated FDC MICs and resistance [31–34]. Mutations in *ftsI*, which encodes for PBP-3, associated with FDC resistance have been documented in strains of *Escherichia coli* and *A. baumannii*. Increased FDC MICs were observed in strains of *E. coli* from a Turkish hospital following YRIN or YRIK insertion after Positions P333 and I532L [35]. YRIN insertion at P333, in combination with NDM and *cirA* mutations, has also been detected in FDC-resistant *E. coli* [36]. Additionally, mutations in PBP-3 (including H370Y) have been detected in FDC-resistant *A. baumannii* isolates, often in combination with various β-lactamases [28].

Studies have showed that multiple mechanisms of resistance, often β-lactamases in combination with permeability defect mutations, appear synergistic, leading to FDC resistance. Lan et al. demonstrated that in isolates of *K. pneumoniae*, the presence of NDM-5 or *cirA* mutations alone led to an eightfold (0.5–4 mcg/mL) and twofold (0.5–1 mcg/mL) increase in FDC MIC, respectfully [37]. When both NDM-5 and *cirA* mutations were introduced, the FDC MIC increased to greater than 256 mcg/mL.

Classified as a multidrug-resistant organism, *E. anophelis* has intrinsic resistance to several β-lactams. *Elizabethkingia* spp. possess two distinct MBLs, *bla*_B_ and *bla*_GOB_, which can be divided into three subclasses: B1, B2, and B3 [14, 15]. *bla*_B_, a subclass B1 MBL, broadly hydrolyzes penicillins, cephalosporins, carbapenems, and novel β-lactam/β-lactamase inhibitors. *bla*_GOB_, a subclass B3 MBL, is hindered by elevated concentrations of *bla*_B_ and is unlikely to contribute to additional β-lactam resistance. Novel MBL subclasses, including *bla*_B−1_, *bla*_B−16_, and *bla*_GOB−19_ have been reported in *Elizabethkingia* spp. [38, 39] *bla*_CME_, intrinsic to *E. anophelis*, confers broad resistance to penicillins and cephalosporins. *E. anophelis* may possess additional resistance mechanisms affecting other antibiotic classes. Fluoroquinolone resistance is primarily mediated by mutations in quinolone resistance determining regions; the most common mutations via amino acid alterations at Positions 83 and 95 in GyrA yielded high-level resistance [40]. Fluoroquinolone-related efflux pump AcrB upregulation has been documented in fluoroquinolone-resistant *E. anophelis* strains [41]. Tetracycline resistance may be mediated by mutations in ribosomal protection proteins *tetB(P)* and *otrA*, and efflux proteins *otrB*, *tetA*, and *tetB* [14]. Additionally, five *E. anophelis* isolates in China conferred pan-tetracycline resistance via *tetX*, a flavin-dependent monooxygenase that leads to enzymatic degradation of tetracyclines [42]. *dfr*A12, *sul* I, *sul* II, and *folP* mutations conferring resistance to SXT are found in *E. anophelis*, although sporadic documentation in SXT-resistant strains leaves their role uncertain [40]. Additionally, isolation of aminoglycoside-inactivating enzymes, *aac*3-*i*, *ant*-6, *ant*-6-*i*, *aac*6, and *aac6-lad*, and *vanW* mutations have removed aminoglycosides and VAN as reliable treatment options [14]. The macrolide, lincosamide, and streptogramin (MLS) resistance genes *ermF*, *ereD*, *mefC*, and *mphG* have been identified in *Elizabethkingia* spp [43].

A positive association between *E. anophelis* biofilm formation and antibiotic resistance has been observed. The analysis of 197 multidrug-resistant *E. anophelis* isolates identified 20 biofilm-positive, extensively drug-resistant isolates. Sputum samples showed the highest percentage of strong biofilm-forming strains (*p* < 0.05), whereas weak biofilms formed mainly in bloodstream infections (*p* < 0.05). Strong–moderate biofilm-forming strains were associated with a statistically significant higher average number of resistances (11.01 ± 0.1643, *p*=0.0006) compared with weak formers (9.657 ± 0.3447), especially for TZP, cefepime, amikacin, and CIP [44].

Yasmin et al. characterized the molecular mechanisms of antimicrobial resistance in an *E. anophelis* isolate resistant to all β-lactams and carbapenems and found it was resistant to CZA, I-R, MVB, and CZA plus ATM combination [1]. FDC, which is stable against hydrolysis by both serine and MBLs, was determined susceptible via BMD (MIC 2 mcg/mL). Additional literature regarding the activity of FDC versus *Elizabethkingia* spp. is extremely limited; two studies reported on four *E. meningoseptica* isolates, all were FDC sensitive with MICs ≤ 4 mcg/mL and ≤ 1 mcg/mL [31, 45].

Although *bla*_B−3_, *bla*_GOB−4_, *bla*_CME−type_, and two single nucleotide polymorphisms in *ftsI* were identified via WGS, the exact mechanism behind FDC resistance in our isolate is unknown, although likely multifactorial. Further WGS, including evaluation of iron transport and porin channels in *E. anophelis*, is necessary to elucidate FDC resistance mechanisms.

## 4. Conclusion

Despite early data suggesting that FDC may be an option for the treatment of *Elizabethkingia* spp. infections, clinicians should exercise caution and obtain susceptibility testing especially when considering FDC monotherapy, considering FDC resistance confirmation in our isolate.

## Figures and Tables

**Table 1:** Antimicrobial susceptibility testing results via broth microdilution (MIC [mcg/mL]).

*Elizabethkingia*⁣*meningoseptica*^1^																						
Drug	AMI	ATM	CFZ	CAZ	CIP	CST	C/T	CZA	FEP	FDC	GEN	IMI	LVX	MEM	MVB	MIN	TZP	TET	TGC	TOB	SAM	SXT
MIC	≥ 64	≥ 64	≥ 64	≥ 64	0.5	—	—	—	≥ 64	≥ 64	≥ 16	≥ 16	0.5	≥ 16	—	1	≥ 128	≥ 16	4	≥ 16	—	2/38
	(R)	(R)	(R)	(R)					(R)	(R)	(R)	(R)		(R)		(R)	(R)	(R)	(R)	(R)		

*Elizabethkingia*⁣*anophelis*^2^																						
MIC	≥ 64	—	—	—	2	> 8	32	32	≥ 64	8	—	—	—	32	32	—	—	—	—	—	32	4
	(R)					(R)	(R)	(R)	(R)	(R)				(R)	(R)						(R)	

*Note:* AMI, amikacin; ATM, aztreonam; CAZ, ceftazidime; CFZ, cefazolin; CIP, ciprofloxacin; CST, colistin; FDC, cefiderocol; FEP, cefepime; GEN, gentamicin; IMI, imipenem; LVX, levofloxacin; MEM, meropenem; MIN, minocycline; R, resistant; SAM, ampicillin-sulbactam; SXT, trimethoprim/sulfamethoxazole; TET, tetracycline; TGC, tigecycline; TOB, tobramycin; TZP, piperacillin-tazobactam.

Abbreviations: C/T, ceftolozane-tazobactam; CZA, ceftazidime-avibactam; MIC, minimum inhibitory concentration; MVB, meropenem-vaborbactam; WGS, whole genome sequencing.

^1^Organism identification and susceptibilities via VITEK 2 ID and AST cards (automated).

^2^WGS and confirmatory susceptibilities performed by IHMA.

## Data Availability

Data sharing is not applicable to this article as no datasets were generated or analyzed during the current study.
